# Hydrodynamic Study of Flow-Channel and Wall-Effect Characteristics in an Oscillating Hydrofoil Biomimetic Pumping Device

**DOI:** 10.3390/biomimetics11010080

**Published:** 2026-01-19

**Authors:** Ertian Hua, Yang Lin, Sihan Li, Xiaopeng Wu

**Affiliations:** College of Mechanical Engineering, Zhejiang University of Technology, Hangzhou 310023, China; 221123020245@zjut.edu.cn (Y.L.); 221123020408@zjut.edu.cn (S.L.); 211123020069@zjut.edu.cn (X.W.)

**Keywords:** flow channel, gap ratio, pumping performance

## Abstract

To clarify how flow-channel configuration and wall spacing govern the hydrodynamic performance of an oscillating-hydrofoil biomimetic pumping device, this study conducted a systematic numerical investigation under confined-flow conditions. Using a finite-volume solver with an overset-grid technique, we compared pumping performance across three channel configurations and a range of channel–wall distances. The results showed that bidirectional-channel confinement suppresses wake deflection and irregular vorticity evolution, enabling symmetric and periodic vortex organization and thereby improving pumping efficiency by approximately 33.6% relative to the single-channel case and by 62.7% relative to the no-channel condition. Wall spacing exhibited a distinctly non-monotonic influence on performance, revealing two high-performance regimes: under extreme confinement (gap ratio h/c= 1.4), the device attains peak pumping and thrust efficiencies of 19.9% and 30.7%, respectively, associated with a strongly guided jet-like transport mode; and under moderate spacing (h/c= 2.2–2.6), both efficiencies remain high due to an improved balance between directional momentum transport and reduced vortex-evolution losses. These findings identify key confinement-driven mechanisms and provide practical guidance for optimizing flow-channel design in ultralow-head oscillating-hydrofoil pumping applications.

## 1. Introduction

In plain river-network regions, the low terrain results in weak hydrodynamic conditions, limited drainage and flood-discharge capacity, and severe water pollution, all of which have long posed significant challenges to water-resource management [1]. Previous studies have shown that combined pump–gate regulation [2,3] can effectively improve water quality and enhance hydrodynamic conditions, offering an important strategy for addressing these challenges. Panda et al. [4] indicates that increasing river discharge accelerates pollutant degradation and thereby enhances the self-purification capacity of the water body. However, conventional pumping stations exhibit low efficiency and poor operational stability under ultralow-head conditions [5,6], and are unsuitable for river channels where the water head is nearly zero. Their operation under such conditions may even exert adverse impacts on the local aquatic ecosystem. By contrast, the flapping-hydrofoil biomimetic pump—a novel water-propulsion device—offers a simple structure, low cost, ease of installation, and high efficiency even under nearly zero-head conditions. These advantages enable it to improve drainage patterns and enhance water-transport efficiency in plain river systems, highlighting its substantial research potential.

As an efficient biomimetic pumping device, the oscillating wing has attracted increasing attention in recent studies for its distinctive hydrodynamic characteristics. Through periodic oscillation, the wing generates a pulsating jet, and such unsteady flow features play a decisive role in determining its pumping performance. Wang et al. [7], using numerical simulations based on the immersed-boundary method, reported that the formation and shedding patterns of vortices in the wake of a non-sinusoidally forced flapping hydrofoil are strongly dependent on the kinematic parameters. The evolution of these vortex structures directly governs momentum transport efficiency and thereby influences the overall pumping performance. Wu et al. [8] demonstrated in their study on centrifugal pumps that suppressing the jet–wake structure can effectively mitigate pressure pulsation. This finding provides an important reference for understanding the pulsating-jet behavior of oscillating wings. Alkhabbaz et al. [9] introduced a hybrid wave energy converter integrating raft- and flap-type concepts and evaluated its performance through experimental and numerical testing.

The influence mechanisms of hydrofoil kinematic parameters on pumping performance are complex and crucial. Systematic numerical studies by Yu et al. [10] showed that within a frequency range of 1–30 Hz, pitch amplitudes of 3–19°, and various nondimensional heave amplitudes, an optimal combination of parameters exists that maximizes propulsion efficiency. More recently, Ding et al. [11] introduced the concept of a frequency–amplitude correlation parameter, which was found to be closely associated with thrust and lift generation, offering a new perspective for optimizing kinematic parameters. Akoz [12] analyzed intermittent propulsion of a combined heaving–pitching foil and discussed the role of reduced frequency in shaping efficiency trends through the coupling of unsteady forces and power input. Liu et al. [13], in their study of passive flexible oscillating-wing energy harvesters, showed that flexible wings can achieve markedly higher energy extraction efficiency than rigid wings within specific stiffness ranges. Fernandez [14] further identified a transition point in thrust generation for semi-flexible hydrofoils, with the optimal bending stiffness yielding efficiency improvements of up to 69%. Recent work by Sun et al. [15] examined how hydrofoil curvature affects the hydraulic performance of flapping-hydrofoil biomimetic pumps, revealing the underlying mechanisms governing the interaction between flexible deformation and vortex structures.

The jet-enhancement mechanisms of oscillating wings within flow channels are key to improving pumping performance. A comparative analysis by Hua et al. [16] showed that out-of-phase oscillation of dual flapping hydrofoils yields significantly superior pumping efficiency compared with in-phase motion. This enhancement arises from the synergistic interactions between the two wings, which optimize vortex pairing and merging processes, leading to increased jet momentum and directionality. Yang et al. [17] used a discrete vortex method to study heave–pitch hydrofoil propulsion, providing a complementary perspective on how kinematic parameters shape thrust/efficiency and wake organization. Numerical investigations by Dryden et al. [18] on biomimetic elastic microvalves provide design guidelines for achieving passive pumping driven by mechanical motions. Despite the difference in functional objectives, propulsion-based pumping and energy harvesting share a common hydrodynamic foundation. Both applications are governed by the unsteady flow physics induced by oscillating hydrofoils, including vortex generation and shedding, wake topology, momentum redistribution, and the interaction between vortical structures and surrounding boundaries. As a result, many studies originally conducted in the context of energy harvesting provide valuable physical insights into wake control, vortex coherence, and flow–structure interaction mechanisms that are directly relevant to understanding propulsion-induced pumping behavior. In particular, previous energy-harvesting studies have extensively examined how oscillation kinematics, frequency–amplitude coupling, and flow confinement influence vortex stability and momentum transport efficiency. These findings offer important references for analyzing how oscillating hydrofoils can effectively impart momentum to the fluid and generate a directed jet, which constitutes the fundamental pumping mechanism investigated in this study.

Flow-channel effects are a key factor governing the hydrodynamic performance of oscillating wings, particularly under confined-flow conditions. Mann et al. [19] investigated the confinement effect on a fully passive oscillating-foil turbine using adjustable sidewalls and reported that strong confinement can alter the vortex formation timing and the effective kinematics, potentially causing an abrupt performance deterioration when the foil approaches the wall. Studies by Liang et al. [20] and Wang et al. [21] on propellers operating near wall boundaries provide important references for understanding flow-channel effects, particularly regarding the evolution of vortex structures in near-wall regions. The mechanisms through which near-wall vortex evolution influences propulsion and pumping efficiency are inherently complex. Li et al. [22] examined the ground-wall effect on a self-propelled flexible foil and showed that near-wall conditions can markedly change the propulsive characteristics and wake topology, providing additional evidence that wall proximity may reshape vortex–jet dynamics. Jiang et al. [23] demonstrated that incorporating a diffuser as a shroud in oscillating-wing turbines can enhance energy-extraction performance. This dual-wall configuration improves the loading on the hydrofoil by guiding the flow and optimizing the pressure distribution. Comparative analyses by Wang et al. [24] further revealed how ducts influence vortex generation, evolution, and interaction, with particular emphasis on the distinctive behavior of tip-leakage vortices in the near-wall region.

Building upon previous research on flapping-hydrofoil devices, this study investigated in depth how different flow-channel widths and configurations affect the pumping performance of flapping hydrofoils. Using two-dimensional numerical simulations complemented by experimental validation, the internal flow fields and hydrodynamic performance of the device under several flow-channel configurations were analyzed. Although flapping-hydrofoil systems have shown great potential in water-propulsion applications, most existing studies focus on optimizing kinematic parameters and hydrofoil geometries, while the influence of flow-channel effects on hydrodynamic performance has been largely overlooked. As a key factor governing the performance of oscillating wings, flow-channel effects become particularly pronounced under confined-flow conditions, directly altering vortex formation and evolution and thereby shaping hydraulic performance. From the perspective of flow-channel effects, this work systematically analyzed and compared the hydrodynamic characteristics under three distinct channel configurations, with particular emphasis on the bidirectional-channel configuration. By varying the wall distance, the optimal pumping performance was further examined. The findings provide important theoretical insights and practical guidance for optimizing the engineering design of flapping-hydrofoil biomimetic pumps and improving their efficiency and stability in real-world applications.

It should be noted that the present study focused exclusively on propulsion-induced pumping mechanisms of oscillating hydrofoils and did not involve energy extraction or energy-harvesting applications. Unlike energy-harvesting systems, in which hydrofoils extract kinetic energy from an incoming flow, the hydrofoil in this study operates as an actively driven device that continuously injects mechanical energy into the fluid through prescribed heaving–pitching motions. The resulting pumping performance is therefore governed by propulsion-induced momentum transfer under confined-flow conditions. The energy-harvesting literature cited in this work is referenced primarily for its contribution to the understanding of oscillating-hydrofoil-induced flow physics, rather than for its application in energy extraction. The present study was strictly concerned with propulsion-driven momentum transfer and pumping performance under flow-channel confinement.

## 2. Physical Model

### 2.1. Motion Description

**Figure 1:**
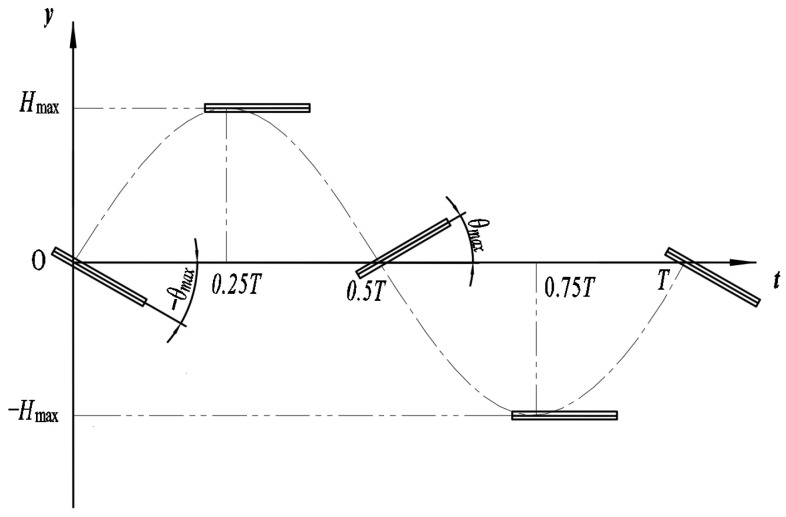
Schematic of the flapping motion of the hydrofoil.

The wall effect refers to the phenomenon in which the presence of solid boundaries imposes impermeability and no-slip conditions on the flow, thereby altering the velocity distribution, shear stress, pressure field, and vorticity structures and ultimately influencing the hydrodynamic performance of nearby moving bodies. In constructing the kinematic model of this study, a flat plate is selected as the fundamental hydrofoil profile. Compared with standard hydrofoils that contain geometric thickness and camber, the flat plate offers simple geometry and well-defined parameters, avoiding interference from curvature and thickness distribution on the hydrodynamic results. This allows the influence mechanisms of wall effects to be elucidated more clearly. In addition, the flat plate is easy to fabricate and convenient to assemble, which enhances experimental repeatability and facilitates numerical validation. Therefore, the flat plate serves as a more suitable baseline model for analyzing flow-channel confinement effects in this study. The flapping flat plate in this work mimics the oscillatory motion of a fish tail and undergoes a two-degree-of-freedom coupled heaving–pitching motion. Following the formulation in Ref. [21], the motion is expressed using standard sinusoidal functions to ensure kinematic continuity and analytical tractability, while also enabling direct comparison with existing studies. In Figure 1, *H_max_* denotes the heave amplitude, *θ_max_* the pitch amplitude, and *T* the oscillation period of the flapping wing.

The fundamental motion equations of the flapping hydrofoil are given as follows [25]:

(1) $$\left\{\begin{array}{c} V_{y}(t)=H_{max}\sin(\omega t)\\ \theta (t)=\theta_{max}\sin(\omega t+\varphi ) \end{array}\right.$$

In the equations, $V_{y}(t)$ denotes the heave displacement of the flapping hydrofoil, θ(t) the pitching displacement, ω the angular oscillation frequency, and φ the phase difference between the heaving and pitching motions.

The velocity expressions of the flapping hydrofoil are given by:

(2) $$\left\{\begin{array}{c} {\dot{V}}_{y}(t)=\omega H_{max}\cos(\omega t)\\ \dot{\theta }(t)=\omega \theta_{max}\cos(\omega t+\varphi ) \end{array}\right.$$

In the equations, ${\dot{V}}_{y}(t)$ represents the heave velocity of the flapping hydrofoil, and $\dot{\theta }(t)$ denotes its pitching velocity.

**Figure 2:**
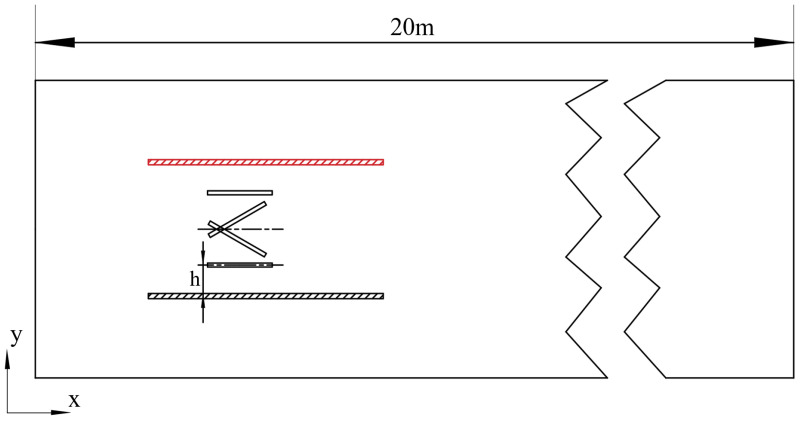
Overall structural schematic.

A flow channel is a confined passage formed by solid boundaries that constrains and directs fluid motion, thereby regulating the transfer of fluid momentum. It represents a key structural component enabling the flapping-hydrofoil pumping device to achieve effective water-pushing performance. To systematically investigate the influence of different flow-channel configurations and wall-distance variations on the hydrodynamic performance of the pumping device, Figure 2 presents schematic diagrams of the overall structures under various flow-channel conditions and wall-distance settings, providing clear geometric and boundary descriptions for subsequent numerical analyses. The nondimensional gap ratio is defined as h/c, where c is the hydrofoil chord length. In this study, the wall distance is categorized into three regimes: extremely narrow spacing (2 ≥ h/c ≥ 1.4), moderate spacing (h/c = 2–3), and wide spacing (h/c ≥ 3). These regimes correspond to three representative flow modes, respectively: jet-dominated flow, high-efficiency cooperative vortex-street flow, and free-diffusion flow.

### 2.2. Hydrodynamic Parameters

To evaluate the hydrodynamic characteristics of the flapping hydrofoil, key performance indicators such as the thrust coefficient, lift coefficient, propulsion efficiency, and pumping efficiency must be quantified. In the nondimensionalization of the kinematic parameters, the Strouhal number is introduced to characterize the unsteady features of the oscillatory motion and its impact on overall hydrodynamic performance. It is defined as $\mathrm{St}=\frac{2fA_{max}}{U_{\infty }}$, where $U_{\infty }$ denotes the freestream velocity and *f* is the oscillation frequency of the hydrofoil [26].

The instantaneous thrust coefficient $C_{T}$, lift coefficient $C_{L}$, and moment coefficient $C_{M}$ of the flapping hydrofoil are as follows [27]:

(3) $$C_{T}(t)=\frac{F(t)}{0.5\rho {\bar{U}}^{2}A}$$

(4) $$C_{L}(t)=\frac{L(t)}{0.5\rho {\bar{U}}^{2}A}$$

In the equations, *F* represents the instantaneous thrust generated by the flapping hydrofoil, *L* represents the instantaneous lift, ρ denotes the fluid density, and *A* represents the projected area of the flapping hydrofoil.

The average thrust coefficient ${\bar{C}}_{T}$ and lift coefficient ${\bar{C}}_{L}$ of the flapping hydrofoil are as follows:

(5) $${\bar{C}}_{T}=\frac{\int_{t}^{t+T}C_{T}(t)dt}{T}$$

(6) $${\bar{C}}_{L}=\frac{\int_{t}^{t+T}C_{L}(t)dt}{T}$$

In the equation, *T* represents the oscillation period.

The formula for the average input power $\bar{P_{a}}$ of the flapping hydrofoil is as follows:

(7) $$\bar{P_{a}}=\frac{\left(\int_{0}^{T}L(t){\dot{V}}_{y}(t)dt+\int_{0}^{T}M(t)\dot{\theta }(t)dt\right)}{T}$$

In the equation, ${\dot{V}}_{y}(t)$ represents the heaving velocity of the flapping hydrofoil, $\dot{\theta }(t)$ represents the pitching angular velocity of the flapping hydrofoil, and M(t) denotes the instantaneous moment generated by the flapping hydrofoil.

To further investigate the propulsion performance of the flapping hydrofoil, parameters such as propulsion efficiency, flow rate and pumping efficiency are introduced.

The formula for the propulsion efficiency η of the flapping hydrofoil is as follows:

(8) $$\eta =\frac{\bar{F_{x}}\bar{U}}{\bar{P_{a}}}$$

In the equation, $\bar{F_{x}}$ represents the average thrust generated by the hydrofoil.

The formula for the average flow rate $\bar{Q}$ at the outlet after stabilization is as follows:

(9) $$\bar{Q}=\bar{U}BA$$

In the equation, *B* represents the river channel width.

The formula for the pumping efficiency $\eta_{pe}$ of the flapping hydrofoil is as follows:

(10) $$\eta_{pe}=\frac{\Delta \bar{P}\bar{Q}}{\bar{P_{a}}}$$

In the equation, $\Delta \bar{P}$ represents the time-averaged total pressure difference between the outlet and inlet of the computational domain. The total pressure is spatially averaged over the entire cross section at each boundary and subsequently averaged over one oscillation period after a statistically periodic state is reached. The inlet and outlet pressure probes are located at distances of 3c upstream and 17c downstream of the hydrofoil pivot, respectively, where *c* is the chord length.

## 3. Materials and Methods

### 3.1. Governing Equations and Turbulence Model

In terms of numerical solutions, this study employs the finite-volume method (FVM) and uses the CFD software Ansys Fluent-2025R2 for flow-field calculations. For incompressible flow, the Reynolds-averaged forms of the mass conservation equation and momentum conservation equation are used as the governing equations, with their specific expressions given as follows [28].

(11) $$\frac{\partial {\bar{u}}_{i}}{\partial x_{i}}=0$$

(12) $$\rho \left(\frac{\partial \bar{\vec{v}}}{\partial t}+\bar{\vec{v}}\cdot \nabla \bar{\vec{v}}\right)=–\nabla \bar{p}+\mu \nabla^{2}\bar{\vec{v}}+\rho \bar{\vec{f}}–\nabla \cdot (\rho \bar{{\vec{v}}_{i}^{\prime }{\vec{v}}_{j}^{\prime }})$$

In the equations, $\rho \bar{{\vec{v}}_{i}^{\prime }{\vec{v}}_{j}^{\prime }}$ represents the Reynolds stress tensor associated with fluctuating momentum, and $–\nabla \cdot (\rho \bar{{\vec{v}}_{i}^{\prime }{\vec{v}}_{j}^{\prime }})$ represents the impact of turbulent fluctuations on the mean flow.

During the heaving–pitching motion, vortex structures are continuously generated and shed from the leading and trailing edges of the flapping hydrofoil. This vortex evolution plays a crucial role in the propulsion performance of the hydrofoil. To more accurately capture the complex flow field involving vortex generation, motion, and shedding, the realizable k−ε turbulence model is chosen for numerical simulations. This model is particularly well suited for flows with pronounced vortical features. The turbulence kinetic energy governing equation is given as follows [29]:

(13) $$\frac{\partial \left(\rho k\right)}{\partial t}+\frac{\partial }{\partial x_{j}}\left[\rho u_{j}\frac{\partial k}{\partial x_{j}}-\left(\mu +\frac{\mu_{t}}{\sigma_{k}}\right)\frac{\partial k}{\partial x_{j}}\right]=\tau_{tij}S_{ij}-\rho \varepsilon +\varphi_{k}$$

The dissipation rate transport equation is as follows:

(14) $$\frac{\partial \left(\rho \varepsilon \right)}{\partial t}+\frac{\partial }{\partial x_{j}}\left[\rho u_{j}\varepsilon -\left(\mu +\frac{\mu_{t}}{\sigma_{\varepsilon }}\right)\frac{\partial \varepsilon }{\partial x_{j}}\right]=c_{\varepsilon 1}\frac{\varepsilon }{k}\tau_{tij}S_{ij}-c_{\varepsilon 2}f_{2}\rho \frac{\varepsilon^{2}}{k}+\varphi_{\varepsilon }$$

In the present study, the working fluid is modeled as an incompressible Newtonian fluid with constant molecular viscosity. This assumption is adopted to isolate the hydrodynamic effects of flow-channel configuration and wall spacing on vortex evolution, confined jet formation, and propulsion-induced pumping performance. We acknowledge that in certain practical scenarios, river water may contain suspended sediments or other constituents that could lead to non-Newtonian behavior. Incorporating non-Newtonian constitutive relations would require additional material parameters as well as application-specific rheological data for calibration and validation. Such extensions are therefore beyond the scope of the present work and will be considered in future studies when reliable rheological information is available.

It is also noted that the present manuscript emphasizes hydrodynamic performance metrics and flow-structure interpretation rather than a full energy-budget decomposition. Although vortex formation and evolution are qualitatively linked to energy losses in the confined channel, a quantitative dissipation analysis—such as computing viscous dissipation rate fields and integrating dissipation over the domain and cycle—would provide a more direct measure of vortex-induced losses. Performing such dissipation-based diagnostics requires additional dedicated post-processing and is therefore left for future work. In subsequent studies, we will quantify confinement-related energy losses using dissipation-rate budgets to further strengthen the mechanistic interpretation of efficiency variations.

### 3.2. Grid Generation and Computational Setup

**Figure 3:**
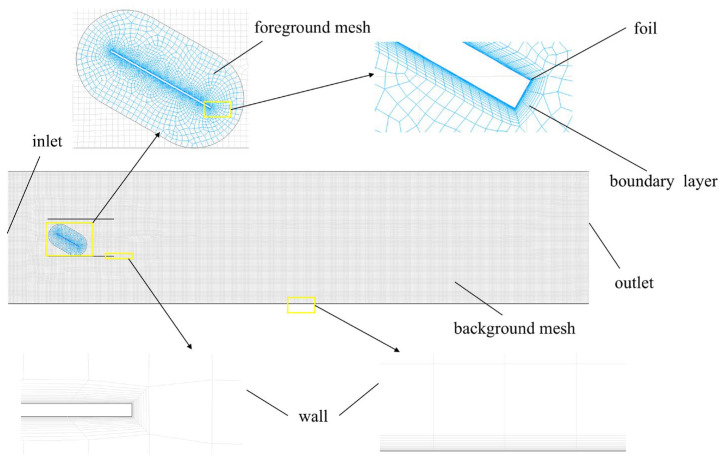
Schematic of grid generation.

To avoid interference from the computational domain setup on numerical results, it is crucial to appropriately determine the boundary location and the scale of the computational region. To ensure the downstream flow field of the flapping hydrofoil is fully developed, the overall domain length is set to 20c in this study. Simultaneously, to balance computational accuracy with cost and avoid mesh distortion during large oscillations, the overset grid method is used to handle the periodic heaving–pitching motion of the hydrofoil. The computational domain consists of a moving foreground grid and a stationary background grid, as shown in Figure 3. The foreground grid uses a hybrid mesh around the hydrofoil surface to capture its local unsteady characteristics, while the background grid employs a structured mesh with a maximum cell size of 30 mm. The fixed domain covers a 5 m × 20 m rectangular river channel, which is used to describe the overall flow-field evolution.

The foreground overset mesh is a body-fitted hybrid grid surrounding the hydrofoil to resolve the unsteady near-body flow during the prescribed heaving–pitching motion. To reduce interpolation errors at the overset interface, the outermost cell size of the foreground mesh is matched to the local background-mesh cell size in the overlap region. The overset region is defined to fully cover the hydrofoil motion envelope, and sufficient overlap is maintained throughout the oscillation cycle to ensure stable donor–receptor interpolation.

Boundary-layer meshes are applied to both the hydrofoil surface and the wall boundaries to ensure near-wall resolution. For the channel wall boundaries, the first-layer thickness is 0.0588 mm, with a growth rate of 1.2 and a maximum of 20 prism layers. For the flow-channel wall boundaries, the first-layer thickness is 0.048 mm, with a growth rate of 1.2 and a maximum of 15 prism layers. These settings ensure smooth transition from the near-wall region to the outer mesh while maintaining adequate resolution of the boundary-layer gradients relevant to confined-flow conditions.

To ensure numerical consistency between the foreground and background grids in the transition region, the cell size of the outer layer of the foreground grid is set equal to that of the background grid, minimizing the impact of mesh scale differences on solution accuracy. Different turbulence models and flow-channel treatments impose varying requirements on the $Y^{+}$ value [30,31]. To accurately resolve the flow structures near the channel and maintain computational stability, boundary-layer meshes are applied both in the flow channel and on the surface of the flapping hydrofoil. Specifically, the first layer mesh height on the surface of the flapping hydrofoil is set to 0.000046 mm, corresponding to a $Y^{+}$ value of 1, to meet the requirements for wall proximity resolution in low-Reynolds-number flow channel treatments.

It is emphasized that the present work does not aim to conduct a boundary-layer rheology or wall-friction-loss analysis. Instead, boundary-layer meshing and a near-wall resolution of $Y^{+}\approx 1$ are enforced to ensure that the wall-adjacent velocity gradients are adequately resolved within the adopted RANS wall treatment, thereby improving the reliability of the predicted confined-flow structures and the resulting pumping-performance trends.

The numerical simulations employ the pressure-based solver in Fluent to solve the Navier–Stokes equations, with pressure–velocity coupling achieved using the coupled algorithm. The momentum equations are discretized using a second-order upwind scheme to improve the accuracy of unsteady flow analysis, while the turbulence kinetic energy and dissipation rate equations are discretized with a first-order upwind scheme to ensure computational stability. The boundary conditions are set as follows: pressure-inlet and pressure-outlet conditions are applied at the inlet and outlet. For the main pumping simulations, pressure-inlet and pressure-outlet boundary conditions were applied with identical reference pressures such that no external pressure difference was imposed across the channel. The resulting flow is generated solely by the oscillatory motion of the hydrofoil, the outer boundary of the foreground grid is specified as an overset type, and the river channel, channel boundaries, and the surface of the flapping hydrofoil are all set to wall conditions. The mesh interaction between the foreground and background grids is achieved using the overset grid technique. The heaving and pitching motion of the flapping hydrofoil is driven by a user-defined function (UDF).

### 3.3. Mesh Independence and Method Validation

To ensure the reliability of the numerical results, a grid independence study was conducted in this research. Grid resolution not only affects the ability to capture flow-field details but also directly determines the computational time cost. Therefore, it is essential to verify its convergence characteristics before solving. In this study, the flapping-hydrofoil operating conditions are set with a chord length of *c* = 1 m, frequency *f* = 1 Hz, heave amplitude *H_max_* = 0.45 m, and pitch amplitude *θ_max_* = 30° as the validation case. Three grid systems of different scales were constructed to evaluate the impact of mesh size on the instantaneous hydrodynamic response.

**Figure 4:**
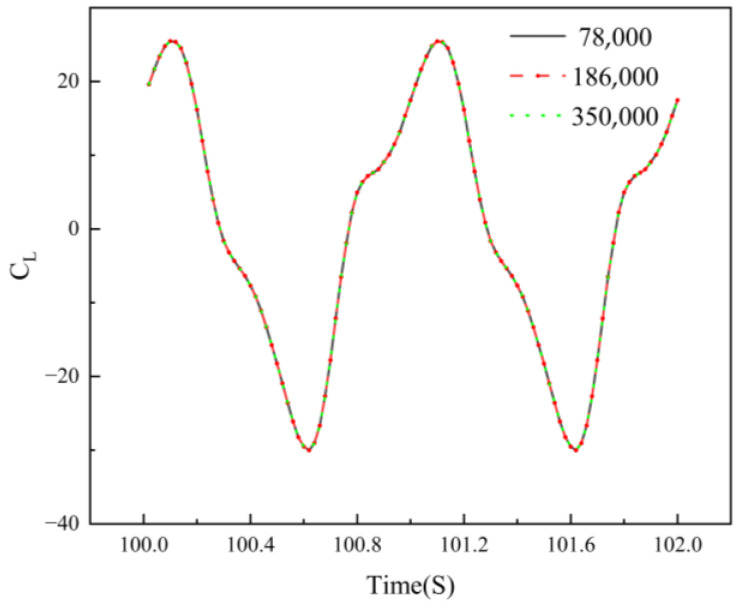
Grid independence validation curve.

The total number of cells for the three grid systems are 78,000, 186,000, and 350,000, respectively, with the corresponding results of the instantaneous thrust coefficient variation over time shown in Figure 4. A comparison reveals that the thrust response curves for all three grid systems are highly consistent, and grid refinement did not lead to significant changes in the results. Although the coarsest grid (78,000 cells) yielded a similar thrust response for the baseline validation case, the medium grid (186,000 cells) was selected for all subsequent simulations to ensure enhanced resolution of complex vortex dynamics and near-wall flows across the wide range of channel configurations and oscillation frequencies investigated in this study.

To validate the effectiveness of the numerical simulation method, the simulation results under identical conditions are compared with the experimental data from Ref. [32].

**Figure 5:**
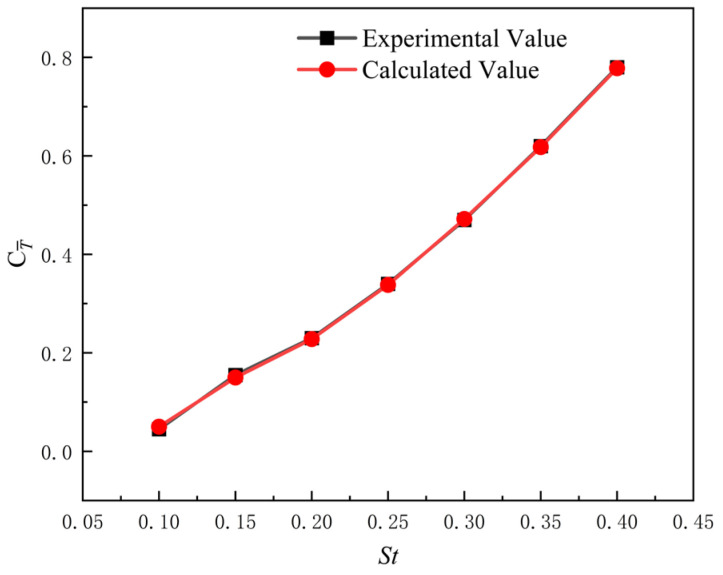
Comparison of numerical and experimental results for the variation in flapping-hydrofoil thrust coefficient at different Strouhal numbers.

The simulation conditions are consistent with the experimental setup in the literature, with parameters such as the chord length c=0.1 m, inlet velocity U=0.4 m/s, phase difference $\phi =\frac{\pi }{2}$, heave amplitude $A_{\max}=0.075$ m, and angle of attack of the biomimetic flapping hydrofoil $\alpha_{\max}=20{}^{\circ}$ matching those used in the experiments. By comparing the numerical results of the average thrust coefficient of the flapping hydrofoil at different frequencies with the experimental data from the literature, as shown in Figure 5, it can be observed that the numerical simulation results are in good agreement with the experimental data. This indicates that the numerical calculation method employed in this study is accurate and effective.

## 4. Results and Discussion

To investigate the impact of the flow-channel structure on the propulsion-induced pumping mechanism and the associated performance trends of the proposed device, the kinematic parameters are fixed as follows for all flapping cases unless otherwise stated: heave amplitude *H_max_* = 450 mm, pitch amplitude *θ_max_* = 30°, and a constant phase difference $\phi =-\frac{\pi }{2}$ between the heaving and pitching motions. With these baseline conditions, the subsequent analyses focus on how variations in flow-channel configuration and wall spacing (gap ratio h/c) regulate the wake/vortex organization, jet formation, and momentum transport within the confined passage, thereby altering the pumping-related metrics such as pressure difference, flow rate, and pumping efficiency.

### 4.1. Influence of Different Flow-Channel Conditions on the Fluid

#### 4.1.1. Impact of Different Flow-Channel Configurations on Thrust and Lift

The thrust and lift coefficients are analyzed separately, as they represent the direct propulsion force and a key indicator of flow symmetry, respectively, both being fundamental to the pumping mechanism.

To investigate the influence of different flow-channel configurations on thrust and lift, calculations were conducted for the corresponding operating conditions at an oscillation frequency of *f* = 1 Hz for three different flow scenarios.

**Figure 6:**
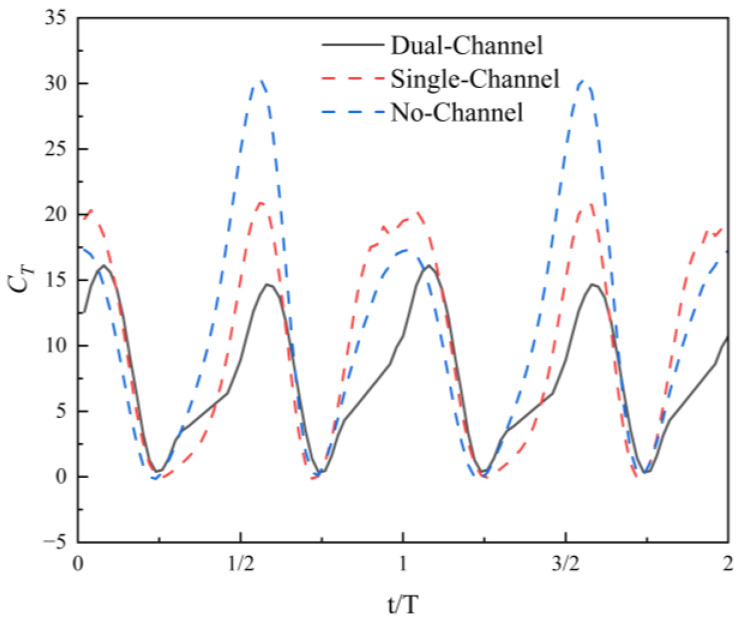
Instantaneous thrust coefficient curve.

Figure 6 presents the variation of the instantaneous thrust coefficient over time for the flapping hydrofoil over two cycles under three different flow-channel conditions: dual channel, single channel, and no channel. It can be observed that the thrust coefficient curves for all three scenarios exhibit good periodicity, indicating that the flow-channel configuration does not alter the phase structure of the forces on the hydrofoil, but significantly affects the peak values and time variations of the thrust coefficient.

Under no-channel conditions, the instantaneous thrust coefficient exhibits the most pronounced periodic fluctuation, with higher peak values and lower trough values. The rise and fall segments of the overall curve change more rapidly, showing the most intense thrust pulsations. This result indicates that in a completely open flow field, the hydrofoil is most sensitive to changes in the freestream, with the largest amplitude of thrust output variation over time. The thrust coefficient curve under single-channel conditions lies between the dual-channel and no-channel cases in terms of both fluctuation amplitude and peak height.

Compared to the no-channel case, the single-channel configuration imposes a certain degree of constraint on the flow in its adjacent region, causing the maximum and minimum thrust coefficient values to converge. However, since the opposite side remains open, the periodic fluctuations of the thrust coefficient are still noticeable, and the overall variation trend maintains a large amplitude. Therefore, it is evident that the single-channel configuration only partially constrains the temporal variation characteristics of the thrust, with its thrust response still exhibiting strong periodicity.

In contrast to the two previous cases, under dual-channel conditions, the instantaneous thrust coefficient curve exhibits lower peak values, minimal fluctuation amplitude, and a smoother shape. The simultaneous presence of flow channels on both sides significantly restricts the flow around the hydrofoil, leading to a more gradual variation in the thrust coefficient over a cycle. Both the rise and fall of thrust over the cycle are more subdued and the overall curve becomes smoother, indicating that the constraint effect of the dual-channel significantly reduces the amplitude of thrust variation over time, leading to more stable thrust output.

The differences in the instantaneous thrust coefficient curves under the three flow-channel conditions exhibit a clear pattern. Under no-channel conditions, thrust variation is most pronounced, with the highest peak value. The single-channel configuration exerts a moderate level of constraint, reducing the amplitude of thrust variation. The dual-channel configuration provides the strongest flow-field constraint, significantly diminishing the periodic fluctuations of the thrust coefficient, resulting in a smoother response. The effect of the flow-channel configuration on the periodic thrust output of the flapping hydrofoil mainly manifests in the adjustment of thrust amplitude and waveform without altering its periodic phase structure.

The differences in the thrust histories under the three channel conditions can be interpreted from the perspective of confinement-induced blockage and pressure recovery. The flow-channel boundaries modify the local acceleration around the hydrofoil and the downstream pressure recovery process, which directly affects the streamwise pressure difference acting on the foil and hence the instantaneous thrust. In the dual-channel case, symmetric confinement provides a more balanced blockage on both sides of the hydrofoil, leading to a more repeatable streamwise pressure-loading pattern and a smoother thrust waveform. In the single-channel case, the one-sided constraint alters the local pressure recovery on the wall-adjacent side, which can intermittently intensify the streamwise pressure difference and produce more pronounced thrust peaks and stronger unsteadiness. Without a channel, the accelerated flow can expand laterally with less restriction, which tends to weaken momentum focusing and increases the variability of pressure loading, resulting in larger thrust fluctuations. From the pumping perspective, a smoother thrust output implies a more stable streamwise momentum input to the confined passage, which is beneficial for maintaining steady pumping performance.

**Figure 7:**
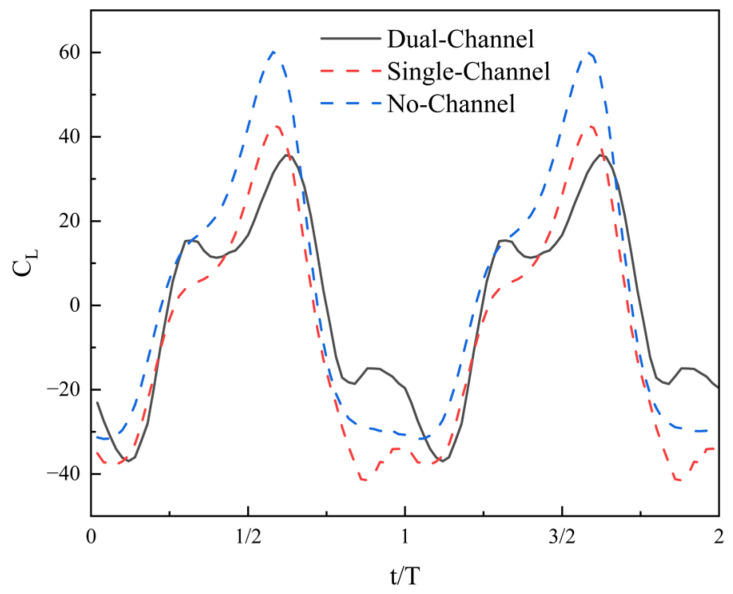
Instantaneous lift coefficient curve.

Figure 7 shows the variation in the instantaneous lift coefficient of the hydrofoil over two oscillation cycles under three different flow-channel conditions The results indicate that the flow-channel conditions primarily affect the temporal distribution characteristics and the cycle-averaged value of the lift coefficient.

Under dual-channel conditions, the lift coefficient exhibits an approximately symmetric distribution around zero throughout the entire cycle, with the positive and negative peaks matching, resulting in a cycle-averaged lift close to zero. This indicates that the symmetric constraint of the dual-side channels leads to a more balanced pressure distribution in the vertical direction, with the positive and negative contributions of lift canceling each other out over one cycle. This feature implies that under dual-channel conditions, the net force in the heaving direction of the hydrofoil is relatively small, with its primary function being the force output in the propulsion direction.

Under single-channel conditions, the positive and negative peaks of the lift coefficient become asymmetric and the distribution above and below zero shifts, resulting in a nonzero cycle-averaged lift. This indicates that the single-sided channel breaks the symmetry of the flow field, increasing the force disparity between the upper and lower surfaces of the hydrofoil and leading to a lift bias. This bias reflects an alteration in the longitudinal force balance of the hydrofoil under single-channel conditions.

Under no-channel conditions, the lift coefficient remains centered around zero, but its temporal variation amplitude is larger than that observed when channels are present. Because the flow field is fully open, the upper and lower flows maintain geometric symmetry, but the lift is more strongly influenced by flow fluctuations, resulting in larger peak-to-peak variations. Although the cycle-averaged lift remains close to zero, both the rate and amplitude of instantaneous lift variation are significantly higher.

The primary influence of flow-channel conditions on lift lies in the cycle-averaged value and distribution symmetry: the dual-channel maintains a symmetric lift distribution around zero, the single-channel introduces a lift bias, and the no-channel condition increases the amplitude of lift fluctuations while preserving symmetry. These characteristics indicate that the flow-channel structure plays a significant regulatory role in the longitudinal force balance of the hydrofoil, providing a foundation for analyzing force stability under different channel configurations. This distinction demonstrates that channel configuration affects not only thrust characteristics but also substantially alters the stability and balance of lift.

The lift histories further reflect how confinement changes the transverse momentum balance and lateral pressure loading. In the dual-channel configuration, the symmetric boundaries tend to constrain lateral flow development on both sides, which helps maintain a more balanced pressure distribution and yields a lift response with reduced bias and improved cycle-to-cycle repeatability. In contrast, the single-channel configuration introduces an inherent lateral asymmetry in blockage and pressure recovery, which manifests as a biased lift response and more uneven positive/negative peaks, indicating a persistent lateral loading tendency. The no-channel case exhibits relatively stronger lift fluctuations, consistent with less constrained lateral flow development and greater variability in transverse loading. These differences are also relevant for practical operation because reduced lift bias implies lower lateral structural loading and improved stability of the pumping device.

#### 4.1.2. Impact of Different Flow-Channel Configurations on Flow Field

In the present pumping configuration, the reverse Kármán vortex street generated by the oscillating hydrofoil does not serve as a propulsion indicator in an open flow, but rather as the primary mechanism for inducing directed momentum transport within the flow channel. The confinement imposed by the channel walls suppresses lateral wake diffusion and stabilizes the streamwise alignment of vortex structures, leading to the formation of a concentrated jet. This jet establishes a stable time-averaged pressure difference along the channel, which directly drives the pumping flow.

During the combined heaving and pitching motion of the oscillating hydrofoil, one side of the foil periodically generates and sheds clockwise vortices, while the opposite side produces counterclockwise vortices. These alternating vortices convect downstream with the flow and form a stable, periodic vortex street. Driven by the continuous motion of the flat plate, the vortices align in the streamwise direction, exhibiting a pattern in which clockwise vortices appear below and counterclockwise vortices appear above. This arrangement, opposite to the classical Kármán vortex street, is known as the reverse Kármán vortex street. The resulting jet-like wake behind the foil generates a thrust that drives the fluid downstream.

To analyze the vortex structures within the flow field, the vorticity component in the z-direction, $\Omega_{z}$, is selected as the characteristic quantity. Its definition is given by:

(15) $$\Omega_{z}=\frac{\partial u_{y}}{\partial x}-\frac{\partial u_{x}}{\partial y}$$

**Figure 8:**
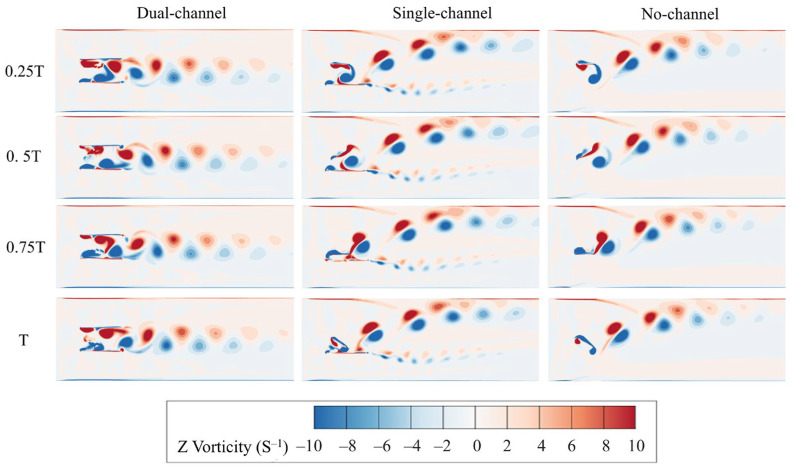
Schematic of the vorticity evolution during four major periods of a cycle under different flow-channel configurations.

Figure 8 illustrates the instantaneous vorticity distribution at various time instants within one oscillation cycle under the dual-channel, single-channel, and no-channel conditions. The results indicate that the flow-channel configuration significantly affects the spatial distribution of vorticity and the formation of the wake. In particular, the dual-channel condition promotes the development of a relatively stable and well-oriented reversed Kármán vortex street.

Under dual-channel conditions, the vorticity is primarily concentrated near the channel center and arranged in a regular pattern along the downstream direction. Throughout the cycle, the generation and shedding of vortex clusters remain consistent and the wake continuously extends along the channel centerline. The spatial extent of vorticity is constrained by the two channel walls, leading to a more concentrated distribution. The spacing and alignment between vortex clusters remain stable, forming a characteristic reversed Kármán vortex street. In the dual-channel configuration, the symmetric confinement on both sides provides balanced blockage and pressure recovery, which suppresses lateral wake deflection and mitigates vorticity offset. As a result, the shed vortices exhibit improved left–right symmetry and a more repeatable cycle-to-cycle organization, and the coherent vortex pattern persists farther downstream with less distortion. This stable and symmetric wake promotes a more aligned, jet-like momentum transport in the streamwise direction, strengthening the net pressure difference and supporting a higher cycle-averaged flow rate and pumping efficiency.

Under single-channel conditions, the vorticity distribution exhibits a pronounced asymmetry. Beginning near the hydrofoil, the fluid on the channel-adjacent side is constrained by the wall, causing local adjustments in velocity and pressure fields. This results in a shear-strength imbalance between the two sides of the hydrofoil, leading the primary reversed Kármán vortex street to develop along the side away from the channel, with its trajectory shifting relative to the geometric centerline. As time progresses, vortex clusters gradually expand in this direction and align along the outer region, forming a continuous biased wake structure. This indicates that the single-channel configuration exerts a persistent influence on the propagation direction of the primary reversed Kármán vortex street, which consistently develops toward the open region.

On the channel-adjacent side, the restricted fluid motion and increased local velocity gradients trigger small-scale flow separation, generating secondary vortex clusters behind the channel wall and forming a small reversed Kármán vortex street. These vortex clusters form immediately downstream of the channel wall, exhibiting a smaller spatial scale compared with the primary wake structure. They are densely arranged, but gradually dissipate as they are convected downstream. This asymmetric vortex organization weakens the effectiveness of momentum focusing into the streamwise direction and introduces additional losses associated with distorted vortex interaction, thereby reducing pumping effectiveness compared with the dual-channel case.

Under no-channel conditions, the vorticity distribution exhibits a freely expanding pattern. The vortex clusters exhibit a relatively uniform intensity within the wake region, and their decay is comparatively slow. The wake expands over a wider region than in the single-channel case, yet no significant directional deviation is observed. The vortex clusters gradually expand downstream, displaying characteristic features of a free flow. Although coherent vortices may still form, their downstream persistence is reduced due to enhanced diffusion and irregular interaction, and the resulting momentum transport is less directionally focused. Compared with the single-channel condition, the vortex clusters in the no-channel case are more symmetrically distributed and widen progressively, whereas those in the single-channel case exhibit a clear lateral bias. Under single-channel conditions, the channel imposes asymmetric constraints on the flow, causing the vortex clusters to shift toward the channel side and producing an asymmetric wake expansion. This phenomenon indicates that the absence of channel walls provides weaker confinement on the vorticity, allowing vortex clusters to expand freely to a larger spatial extent.

#### 4.1.3. Impact of Different Flow-Channel Configurations on Propulsion Performance

To investigate the influence of different flow-channel conditions on propulsion performance, the flapping frequency was varied from 0.2 Hz to 1 Hz in increments of 0.2 Hz and subsequently increased in steps of 1 Hz up to a maximum frequency of 5 Hz.

**Figure 9:**
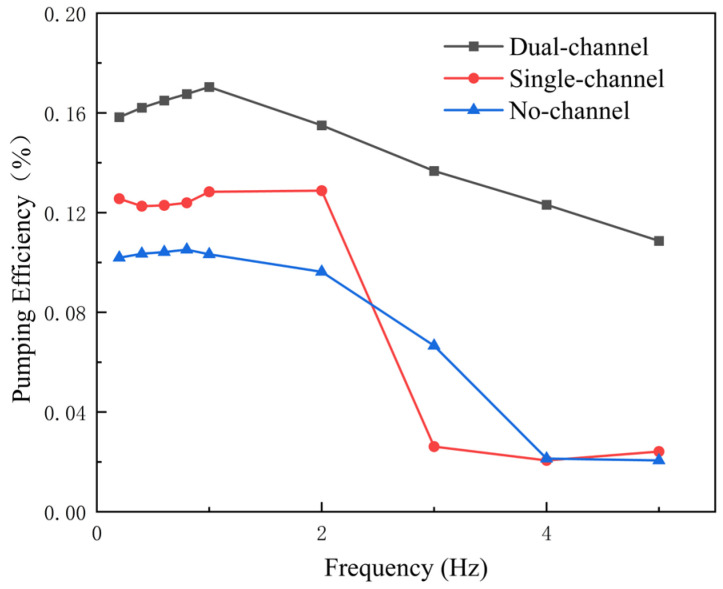
Pumping efficiency curves for three flow-channel configurations at different frequencies.

Figure 9 illustrates the variation in pumping efficiency with frequency under the three flow-channel conditions. In the low-frequency range of 0.2–1 Hz, all three flow-channel conditions exhibit a trend in which the pumping efficiency increases initially and then decreases. Under the dual-channel condition, the pumping efficiency is relatively high at low frequencies, but it gradually decreases as the frequency increases, reaching its minimum value of 10.87% at f=5 Hz. This trend suggests that at low frequencies, the confinement effect provided by the dual channel enhances pumping efficiency; however, as the frequency increases, the flow becomes less stable, resulting in a reduction in pumping efficiency.

Under the single-channel condition, the overall pumping efficiency is relatively low and decreases rapidly with increasing frequency, reaching 2.42% at f=5 Hz. The single-channel configuration constrains the flow on only one side, lacking the symmetric confinement provided by the dual-channel configuration. As a result, this asymmetric confinement disrupts the balance of the flow, causing unequal loading on the upper and lower surfaces of the hydrofoil, which in turn reduces the pumping efficiency. As the frequency increases, flow instability and recirculation effects intensify, further contributing to the decline in efficiency. At higher frequencies, the asymmetry in the flow becomes more pronounced, leading to an even more significant reduction in efficiency under the single-channel condition.

Under the no-channel condition, the pumping efficiency is the lowest at low frequencies and continues to decrease as the frequency increases, reaching 2.06% at f=5 Hz. As the frequency increases, the vortex clusters behind the hydrofoil become more displaced, resulting in a more dispersed wake structure. In the absence of channel confinement, the vortex clusters freely expand in the streamwise direction rather than remaining concentrated within a confined region, thereby reducing the directionality of the jet and ultimately diminishing the pumping efficiency.

The dual-channel configuration exhibits the highest pumping efficiency, particularly in the low-frequency range, where its performance is markedly better than that of the single-channel and no-channel conditions. Compared with the single-channel case, the dual-channel configuration increases pumping efficiency by approximately 33.6%, and compared with the no-channel case by about 62.7%. This indicates that the degree of flow confinement imposed by the channel structure directly affects pumping efficiency. The dual-channel configuration symmetrically constrains the fluid motion, enabling it to deliver optimal pumping performance at low frequencies.

**Figure 10:**
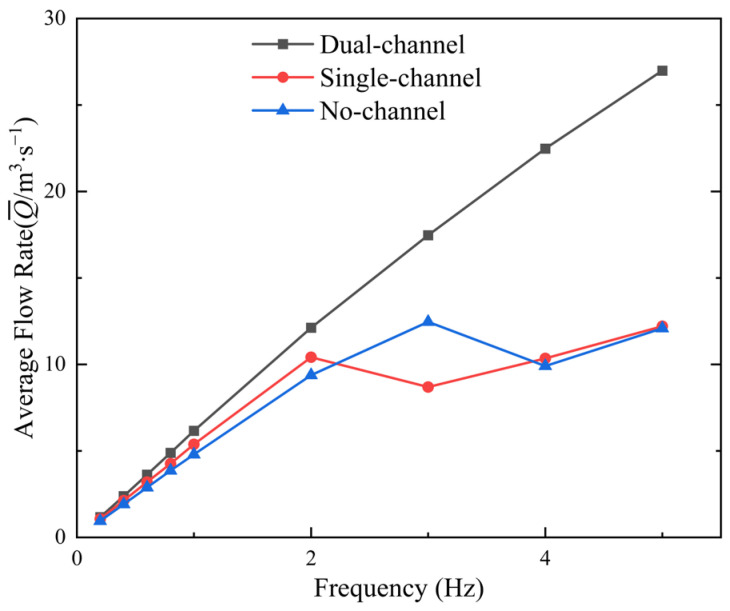
Average flow rate curves for three flow-channel configurations at different frequencies.

Figure 10 illustrates the variation in average flow rate with frequency under the three different flow-channel conditions. In the low-frequency range, the dual-channel condition yields the highest average flow rate, which continues to increase with frequency. The single-channel condition results in a lower average flow rate with a slower rate of increase. Under the no-channel condition, the average flow rate is relatively high at low frequencies, but stabilizes and then decreases as the frequency increases. Enhanced flow confinement significantly improves the average flow rate, and the dual-channel configuration demonstrates a markedly superior flow-acceleration effect compared with the single-channel and no-channel cases, especially at higher frequencies.

### 4.2. Influence of Flow-Channel Wall Spacing on the Fluid

#### 4.2.1. Impact of Flow-Channel Wall Spacing on the Flow Field

In this study, a flapping frequency of f=1 and a pitching amplitude of $\theta_{0}=30{}^{\circ}$ are used as the motion conditions. At t/T=0.5, the gap ratio h/c between the pivot and the flow channel is varied to explore the optimal propulsion strategy. The minimum wall spacing is set to the heave amplitude of the hydrofoil plus 0.2c, ensuring that the hydrofoil does not contact the channel during oscillation. Therefore, the minimum gap ratio is set to h/c=1.4 to prevent trailing-edge contact with the channel. The wall spacing is then gradually increased in increments of 0.2 m up to 4 m.

**Figure 11:**
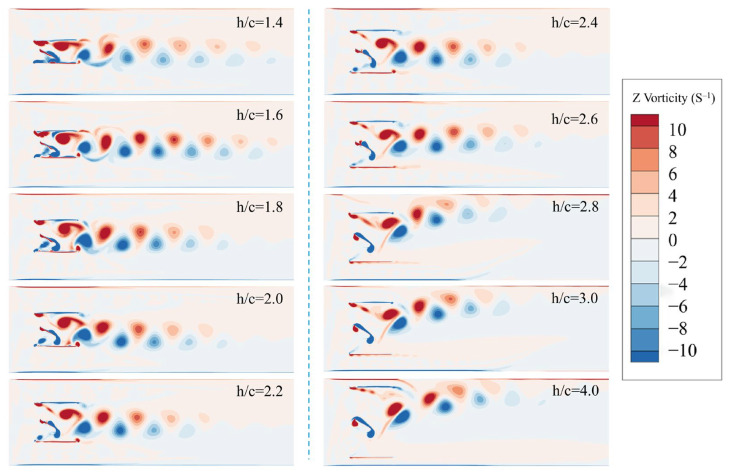
Vorticity contours at different wall spacings.

Figure 11 presents the vorticity contours illustrating the influence of different wall spacings on the flow field. When the gap ratio is in the range of h/c=2.8 to 4, the confinement imposed by the channel weakens and its guiding effect on the vorticity gradually diminishes. As the flow space around the hydrofoil increases, noticeable vortex cluster displacement and spreading begin to occur. During this stage, the principal axis of vorticity is no longer aligned with the channel center, but shifts toward the side away from the channel, leading to a loss of symmetry in the vorticity distribution.

When the gap ratio is in the range of h/c=2.0 to 2.6, the vorticity structures exhibit a clear and extensive reversed Kármán vortex street. This indicates that the motion of the hydrofoil dominates the flow-field formation, with only a minor influence from the channel walls. At this stage, the vortex clusters are relatively dispersed and the wake spreads over a wider region, demonstrating characteristics of a typical free-flow state. The vortex street remains clearly symmetric, and the flow field is relatively stable and largely unaffected by channel confinement.

When the gap ratio falls within the critical range of h/c=1.6 to 1.8, the flow channel begins to exert a significant modulation effect on the flow. Although the vortex street retains partial symmetry, the vortex core undergoes noticeable compression and intensification, resulting in a marked increase in vorticity concentration. The channel between the hydrofoil and the wall accelerates the fluid, strengthening the vortex clusters, which is reflected by the deepened colors of the vorticity contours. This phenomenon indicates that the channel effect has begun to noticeably influence the flow, yet it has not fully suppressed the formation of the wake vortex structures.

At a gap ratio of h/c=1.4, the vorticity intensity is reduced compared with that at h/c=1.6. The vortex clusters in the wake are formed in a more compact manner; however, compared with cases of larger wall spacing, the vorticity is more concentrated and the downstream expansion range behind the hydrofoil is smaller. Channel confinement prevents the vorticity from fully developing as it does at larger wall spacings, thereby weakening the vortex structures. Due to the restricted flow space, the vortex clusters form rapidly, but their downstream spreading is inhibited by the confinement effect.

In this study, vorticity fields are adopted as the primary visualization diagnostic because they directly reveal wake organization, symmetry preservation/breaking, and jet formation, which constitute the dominant mechanism controlling the pumping-related performance trends under different confinement conditions. Shear- or stress-based fields can serve as complementary diagnostics to support a more quantitative loss interpretation, but they are not essential for establishing the mechanism–performance linkage emphasized in the present work. Such stress/dissipation-based visualizations will be considered in future studies dedicated to loss decomposition.

#### 4.2.2. Impact of Flow-Channel Wall Spacing on Propulsion Performance

**Figure 12:**
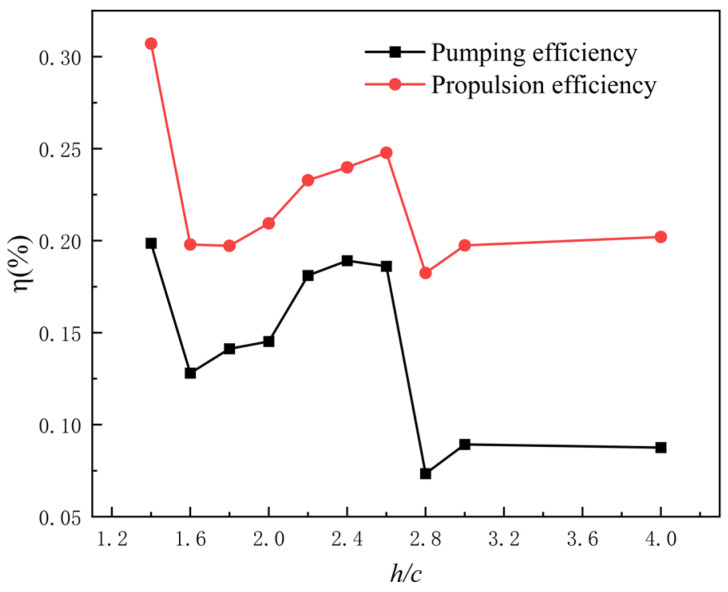
Efficiency curves at different wall spacings.

Figure 12 illustrates the variation trends of pumping efficiency and propulsion efficiency under different flow-channel wall spacings. It can be observed that both efficiencies reach their peak at a gap ratio of h/c=1.4, yielding 19.9% for pumping efficiency and 30.7% for propulsion efficiency. In conjunction with the vorticity distributions presented earlier, the flow field undergoes a structural transition whereby large-scale periodic vortex streets are suppressed and replaced by a strongly confined jet-like flow. This transition shifts the thrust-generation mechanism from one dominated by vortex-induced pressure gradients to a more direct, quasi-steady process driven by instantaneous momentum transfer. By effectively bypassing the substantial energy losses associated with vortex formation and evolution, the system achieves its highest energy-conversion efficiency.

At gap ratios of h/c=1.6 to 2.0, both the pumping efficiency and propulsion efficiency remain at relatively low levels. In conjunction with the vorticity contours, which show the development and strengthening of the vortex street, this corresponds to a sharp decline in efficiency. This indicates that the intensified large-scale vortex structures are inherently associated with substantial energy dissipation. The processes of vortex formation, evolution, and dissipation consume the kinetic energy of the fluid, preventing it from being effectively converted into directed momentum and thereby reducing the overall system efficiency.

At gap ratios of h/c=2.2 to 2.6, both the pumping efficiency and propulsion efficiency increase and remain stable. In conjunction with the vorticity contours, the vortex structures exhibit moderately reduced intensity and diminished flow confinement, leading to a recovery in efficiency. This phenomenon indicates that within this range of gap ratios, the channel effect reaches a balanced state: it neither excessively suppresses the formation and evolution of vortex structures nor induces excessive energy dissipation. Moderate flow-channel confinement effectively guides the flow and reduces the disorderly spreading of vorticity, thereby enhancing the conversion efficiency of fluid kinetic energy and improving the overall system performance.

At a gap ratio of h/c=2.8, the pumping efficiency and propulsion efficiency reach their minimum values, 7.3% and 18.2%, respectively. Moreover, as the wall spacing increases, both efficiencies tend to stabilize at relatively low levels. According to the vorticity contours, the weakening confinement of the channel causes the vortex structures to become more diffuse and less intense, indicating that excessive channel spacing prevents the flow energy from being effectively concentrated. As the wall spacing increases, the wake vortices gradually shift and spread, reducing the fluid kinetic energy-conversion efficiency, which suggests the existence of an optimal range of channel spacing.

**Figure 13:**
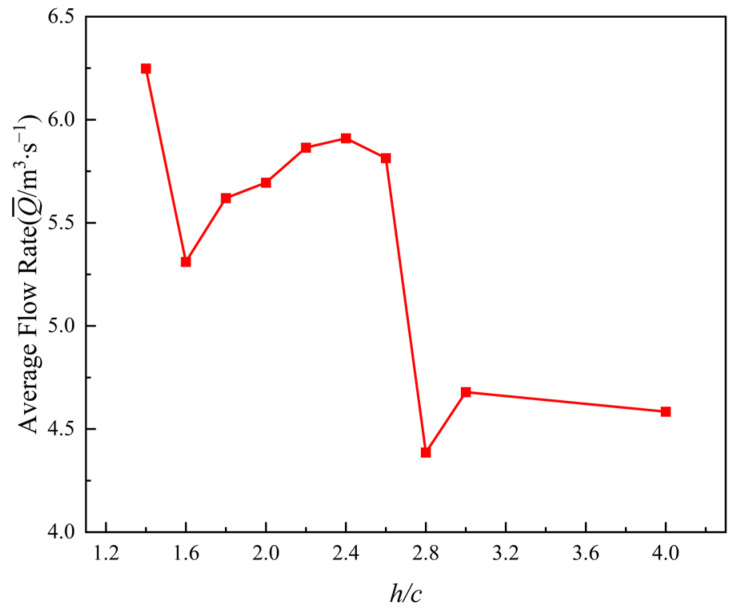
Average flow rate curves at different wall spacings.

Figure 13 presents the curve of average flow rate under different wall spacings. As shown, the average flow rate exhibits a clear non-monotonic variation with wall spacing, indicating a pronounced modulation of flow flux by channel confinement. At the minimum gap ratio of h/c=1.4, the average flow rate reaches its maximum value of 6.25 m^3^/s. Under this condition, the flow enters a near-wall jet-like regime in which strong channel confinement concentrates momentum within the passage, minimizes flow losses, and directly converts input energy into fluid momentum, thereby achieving the highest transport capacity. When the gap ratio increases to h/c=1.6, the average flow rate drops immediately to 5.31 m^3^/s. This indicates that as channel confinement weakens, the integrity of the jet structure deteriorates, allowing fluid momentum to spread laterally and thereby reducing the effective flow flux.

To further clarify the sharp variation in average flow rate observed between h/c=1.4 and h/c=1.6, the flow structures in this narrow-gap regime are examined in more detail. At h/c=1.4, the hydrofoil operates under an extreme near-wall confinement condition, where the flow passage between the hydrofoil and the channel wall behaves as a strongly constrained jet duct. In this regime, lateral flow diffusion is effectively suppressed, and the momentum injected by the oscillating hydrofoil is predominantly redirected into a streamwise jet-like flow. As a result, the wake vortices are highly compressed, their downstream spreading is inhibited, and the flow remains strongly aligned with the channel centerline, leading to a maximum outlet flow rate.

When the gap ratio increases slightly to h/c=1.6, the confinement strength is reduced sufficiently to induce a qualitative change in the near-wall flow topology. The previously compact jet begins to expand laterally, accompanied by enhanced entrainment of surrounding fluid and the emergence of localized recirculation and shear-layer instability near the wall-side gap. This transition weakens the coherence of the jet-like structure and increases viscous dissipation associated with vortex deformation and partial breakdown. Consequently, although the geometric change in wall spacing is small, the effective streamwise momentum transport is significantly reduced, resulting in the pronounced non-linear decrease in the cycle-averaged flow rate.

This behavior indicates that the narrow-gap regime (h/c=1.4–1.6) corresponds to a sensitive transition zone between a strongly confined jet-dominated flow and a less constrained, vortex-influenced transport mode, rather than a gradual geometric scaling effect.

Within the gap ratio range of h/c=1.8 to 2.6, the average flow rate gradually increases with wall spacing and stabilizes between 5.6 and 5.9 m^3^/s, forming a relatively steady flux plateau. The vorticity contours show that within this gap ratio range, the flow develops a relatively well-organized reverse Kármán vortex street. The channel-induced modulation of vortex shedding prevents the wake vortices from being overly compressed or excessively diffused, thereby ensuring efficient downstream momentum transport and maintaining the flow rate at a high level.

When the gap ratio further increases to h/c=2.8, the average flow rate drops sharply to 4.39 m^3^/s, the lowest value across the entire range. At this stage, the wake vortices become noticeably offset and diffuse, indicating an almost complete loss of channel confinement. The momentum streamlines lose their directionality, resulting in a substantial reduction in flow flux. At larger gap ratios of h/c=3 to 4, the average flow rate remains in the lower range of 4.58–4.68 m^3^/s, further indicating that the flow approaches a free-wake state, lacking the momentum-focusing effect normally provided by channel guidance.

## 5. Conclusions

Based on the numerical simulations, this study systematically investigated how flow-channel configuration and wall spacing regulate the pumping mechanism and performance of an oscillating-hydrofoil device under confined-flow conditions. The main conclusions are summarized as follows.

1. Channel confinement significantly modifies the thrust and lift characteristics of the flapping hydrofoil by altering the vorticity distribution and flow symmetry, thereby directly affecting flow-field stability and overall hydrodynamic performance.
2. Among the three channel configurations, the dual-channel arrangement most effectively suppresses wake deflection and vorticity offset, leading to the most stable vortex organization and the best pumping performance. Quantitatively, the peak pumping efficiency of the dual-channel case is improved by approximately 33.6% relative to the single-channel configuration and by about 62.7% relative to the no-channel condition.

The influence of channel spacing on pumping performance exhibits a non-monotonic trend. Rather than a single “optimal” spacing, the low-head pumping device operates efficiently under two distinct performance regimes:

3. Under the extreme near-wall condition with a gap ratio of h/c=1.4, the system achieves its optimal overall performance. In this regime, the channel effect exerts a pronounced modulation on the flow, suppressing periodic vortex structures and establishing a strongly confined jet-like flow. This configuration yields the highest pumping and propulsion efficiencies, 19.9% and 30.7%, respectively. Such a mode is well suited for applications requiring maximal flow rate and efficiency.
4. When the gap ratio lies within h/c=2.4 to 2.6, the channel effect does not suppress vorticity generation, but rather effectively guides and concentrates it. In this regime, the flow dynamics are predominantly governed by the oscillation of the flapping hydrofoil. Both the pumping and propulsion efficiencies remain at relatively high levels within this spacing range. This flow mode achieves an optimal balance between enhancing flow directionality and minimizing energy losses associated with vortex evolution.

It should be noted that the present study is based on two-dimensional numerical simulations. While the 2D framework effectively captures the primary flow mechanisms associated with oscillating-hydrofoil-induced pumping—including vortex generation, confinement-modulated jet formation, and momentum transfer—it inherently neglects three-dimensional effects. In practical pumping devices employing low-aspect-ratio hydrofoils, spanwise flow, tip vortices, and associated tip losses may introduce additional energy dissipation and alter the quantitative performance characteristics. Therefore, although the qualitative trends and physical mechanisms identified in this study are expected to remain valid, the absolute values of pumping and propulsion efficiencies may differ in fully three-dimensional configurations. Future work will focus on extending the present analysis to three-dimensional numerical simulations and experimental investigations to systematically quantify tip-loss effects and further enhance the applicability of oscillating-hydrofoil pumping devices in realistic confined-channel environments.

To contextualize the peak pumping efficiency reported in this study (maximum $\eta_{p}=19.9\%$ at h/c=1.4), it is useful to compare it with conventional turbomachinery-based solutions used in drainage and circulation systems. Axial-flow pumps can reach high peak hydraulic efficiencies under their design conditions (often on the order of 80% or higher), whereas their performance may degrade substantially under ultralow-head or near-zero-head operating regimes due to enhanced flow losses and unsteady dissipation. In contrast, the oscillating-hydrofoil device considered here is intended for ultralow-head environments and achieves pumping by propulsion-induced momentum transfer under confined flow conditions. It should be emphasized that the efficiency reported in this work is a hydrodynamic metric defined by Equation (10), and it does not include system-level losses such as transmission/actuator efficiency and mechanical/electrical conversion losses.

Therefore, the present results are best interpreted as demonstrating the feasibility and optimization trends of a biomimetic pumping mechanism in ultralow-head scenarios, while future work is needed to quantify full system efficiency and practical viability through three-dimensional simulations, prototype testing, and drive-system integration.

## Data Availability

All data, models, and code generated or used during the study appear in the submitted article.

## Abbreviations

The following abbreviations are used in this manuscript.

*H_max_*	heave amplitude
*θ_max_*	pitch amplitude
*T*	oscillation period of the flapping hydrofoil
$V_{y}(t)$	heave displacement of the flapping hydrofoil
θ(t)	pitching displacement
ω	angular oscillation frequency
φ	phase difference between the heaving and pitching motions
${\dot{V}}_{y}(t)$	heave velocity of the flapping hydrofoil
$\dot{\theta }(t)$	pitching velocity of the flapping hydrofoil
$C_{T}$	instantaneous thrust coefficient
$C_{L}$	instantaneous lift coefficient
${\bar{C}}_{T}$	average thrust coefficient
${\bar{C}}_{L}$	average lift coefficient
$\bar{P_{a}}$	average input power of the flapping hydrofoil
η	propulsion efficiency
$\bar{F_{x}}$	average thrust generated by the hydrofoil
$\bar{Q}$	average flow rate
$\eta_{pe}$	pumping efficiency
$\Delta \bar{P}$	pressure difference between the average inlet and average outlet pressures of the flow channel
